# Letter to the editor regarding “Recent intake of direct oral anticoagulants and acute ischemic stroke: real world data from a comprehensive stroke center” by Pommeranz et al.

**DOI:** 10.1186/s42466-026-00463-x

**Published:** 2026-02-03

**Authors:** Matija Zupan, Pawel Kermer, Senta Frol

**Affiliations:** 1 Department of Vascular Neurology, Faculty of Medicine, Ljubljana University, Zaloška 2, Ljubljana, 1000 Slovenia; 2 Faculty of Medicine, University of Ljubljana, Ljubljana, Slovenia; 3 Department of Neurology, Nordwest-Krankenhaus Sanderbusch, Friesland Kliniken GmbH, Sande, Germany; 4 Department of Neurology, University Medical Center Göttingen, Göttingen, Germany

Dear Editor,

With great interest we read the article by Pommeranz et al., titled “Recent intake of direct oral anticoagulants and acute ischemic stroke: real–world data from a comprehensive stroke centre” [1]. We would like to congratulate the authors for providing a comprehensive and clinically relevant contribution to the growing evidence of intravenous thrombolysis (IVT) in acute ischaemic stroke (AIS) patients with recent (< 48 h) direct oral anticoagulant (DOAC) intake.

In their retrospective cohort of 469 AIS patients with documented recent DOAC ingestion and calibrated anti-factor IIa/Xa measurement, the authors report that 28% had anti-factor IIa/Xa activity ≤ 30 ng/ml, 27% >30 – ≤ 100 ng/ml and 45% > 100 ng/ml [1]. Importantly, lower DOAC plasma levels correlated inversely with stroke severity [1]. Among patients with DOAC levels ≤ 50 ng/ml, IVT was administered in 33.5%, whereas among those with levels > 50 ng/ml only 4% received IVT (the majority preceded by reversal with idarucizumab). Symptomatic intracranial haemorrhage (sICH) occurred in 4% of IVT-treated and 1% of non-IVT-treated patients, without statistically significant association with the level of anticoagulation [1]. IVT appeared safe across all DOAC-level strata.

This study significantly adds to the existing evidence in several ways. Historically, patients on DOACs have been excluded from pivotal thrombolysis trials. European Stroke Organisation guidelines recommend against IVT in the absence of reliable coagulation assays with DOAC intake within 48 h prior to AIS [2]. Earlier large-scale observational data from the US cohort including 2,207 thrombolysed AIS patients on DOACs showed that DOAC use within prior 7 days did not significantly increase the risk of sICH (adjusted OR 0.88, 95% CI 0.70–1.10) compared with no anticoagulation [3]. Furthermore, an international retrospective cohort including 832 thrombolysed AIS patients despite documented DOAC ingestion within 48 h found lower odds of sICH compared with no anticoagulant use (adjusted OR 0.57; 95% CI 0.36–0.92) [4]. Among the DOAC-group, 30.3% received reversal (idarucizumab), 27.0% had DOAC-level measurements, and 42.7% received IVT without measurement or reversal. In the DOAC group, sICH rate was 2.5% versus 4.1% in controls. These findings held across the different selection strategies (reversal, levels measured, or neither) and sensitivity analyses of detectable plasma-levels or very recent ingestion. The authors found no evidence of higher sICH incidence associated with IVT in selected AIS patients with recent DOAC ingestion. They suggest that, under certain selection criteria, IVT may be safe in this subgroup, and call for prospective trials.

The Essen cohort now complements that by providing detailed DOAC plasma-level stratification, local workflow data, and real‐world IVT use patterns – highlighting that despite low anticoagulant activity a substantial proportion of eligible patients remain untreated. Equally important, the authors’ finding that a large subset (≈ 28%) of patients with recent DOAC ingestion actually had minimal or no measurable anticoagulant activity underscores the pharmacokinetic/pharmacodynamic variability in real-world DOAC use and possibly, supports the argument for a more liberalised approach to reperfusion therapies in this setting [1]. It is also reassuring to note the low overall sICH incidence (2% in the whole cohort; 4% in IVT‐treated) and lack of association with anticoagulant level group [1]. These observations reinforce the plausible safety of IVT in highly selected patients on DOACs — a crucial point in the design of upcoming prospective trials, including patients with highest levels of DOACs (i.e., > 100 ng/ml) and those being treated with tenecteplase, now widely adopted as the preferred thrombolytic in AIS.

Of note, the ongoing multicentre prospective randomised trial “DO‑IT – The DOAC Intravenous Thrombolysis Trial” (NCT06571149) [5] investigates the safety and efficacy of IVT (with alteplase or tenecteplase) in AIS patients with recent DOAC ingestion (< 48 h), with the primary outcome being a 90-day shift in modified Rankin Scale. The results of DO-IT will be pivotal to definitively inform guideline updates.

In conclusion, we again commend Pommeranz and colleagues for their excellent work and for further advancing the evidence on IVT in AIS patients with recent DOAC intake. Their observations should encourage stroke centres to refine institutional protocols, promote rapid DOAC-level testing workflows, and perhaps most importantly, support broader inclusion criteria of DOAC-treated patients into reperfusion therapy discussions. Looking ahead, we envisage that further large-scale randomised trials and international registries will refine risk-benefit modelling, clarify safe anticoagulant-activity thresholds (or obviate the need for such measurement), and ultimately enable more equitable access to reperfusion therapies in this growing patient subgroup.

## Data Availability

Not applicable.
